# Detection and Removal of Biases in the Analysis of Next-Generation Sequencing Reads

**DOI:** 10.1371/journal.pone.0016685

**Published:** 2011-01-31

**Authors:** Schraga Schwartz, Ram Oren, Gil Ast

**Affiliations:** Department of Human Molecular Genetics and Biochemistry, Sackler Faculty of Medicine, Tel-Aviv University, Ramat Aviv, Israel; Université Paris Sud, France

## Abstract

Since the emergence of next-generation sequencing (NGS) technologies, great effort has been put into the development of tools for analysis of the short reads. In parallel, knowledge is increasing regarding biases inherent in these technologies. Here we discuss four different biases we encountered while analyzing various Illumina datasets. These biases are due to both biological and statistical effects that in particular affect comparisons between different genomic regions. Specifically, we encountered biases pertaining to the distributions of nucleotides across sequencing cycles, to mappability, to contamination of pre-mRNA with mRNA, and to non-uniform hydrolysis of RNA. Most of these biases are not specific to one analyzed dataset, but are present across a variety of datasets and within a variety of genomic contexts. Importantly, some of these biases correlated in a highly significant manner with biological features, including transcript length, gene expression levels, conservation levels, and exon-intron architecture, misleadingly increasing the credibility of results due to them. We also demonstrate the relevance of these biases in the context of analyzing an NGS dataset mapping transcriptionally engaged RNA polymerase II (RNAPII) in the context of exon-intron architecture, and show that elimination of these biases is crucial for avoiding erroneous interpretation of the data. Collectively, our results highlight several important pitfalls, challenges and approaches in the analysis of NGS reads.

## Introduction

The emergence of next-generation sequencing technologies has given an immense boost in the field of DNA sequencing. The major advance offered by NGS is the ability to produce an enormous volume of data relatively cheaply. Several NGS systems are currently on the market, the most widely used one being Illumina. Collectively, they rely on various strategies for template preparation, sequencing, and imaging, followed by genome alignment methods and downstream analysis of the data (reviewed in [1]). The applications of this technology are limited by imagination only: Common applications of the NGS technology are genome assembly [2], identification of structure variants or single nucleotide polymorphisms (SNP)[3], [4], cataloging of the transcriptome (RNA-seq) [5], [6], mapping transcription factor binding sites (ChIP-seq) [6] or sites bound by RNA-binding proteins (CLIP-seq) [7], [8], and genome-wide profiling of epigenetic marks and chromatin structure (e.g., ChIP-seq, methyl-seq, and DNase-seq) [9]–[13].

Since the launching of deep-sequencing technology, enormous efforts have been put into the development of platforms for mapping short reads and for downstream analysis of mapped reads (reviewed in [1], [2], [6]). In parallel, efforts have been made to understand and overcome the biases inherent in the NGS technology [14]–[18]. In this study, we begin by presenting two biases which we encountered in the analysis of NGS reads generated via the Illumina Genome Analyzer. We show the general relevance of these biases across different datasets, when applicable, and demonstrate how failing to normalize for these biases can potentially lead to spurious conclusions. We then demonstrate how both these biases are manifested, and can partially be normalized, in analyzing an NGS dataset mapping transcriptionally engaged RNA polymerases in the context of exon-intron architecture. In the latter dataset we also discovered two additional biases that were present in it, and that are presumably more specific to this particular dataset. Collectively, our results demonstrate that in analyzing a specific NGS dataset, it is necessary to both take into account general biases that are prevalent, or even inherent, in such data, but also to carefully assess the presence of experiment-specific biases in the dataset, and to tailor specific approaches to address such biases and avoid misinterpretation of the data.

## Results

We were first interested in evaluating the general impact of two biases we encountered in the context of analyzing next-generation sequencing datasets: nucleotide per cycle bias and mappability bias.

### Nucleotide per cycle bias

While analyzing different NGS datasets, we observed that distributions of the sequenced nucleotides changed across the positions of the reads. To explore the prevalence of this bias across different deep-sequencing datasets, we analyzed the nucleotide distribution among reads from 25 different lanes of deep-sequencing data in which either genomic DNA or cDNA was sequenced. Specifically, we analyzed RNA-seq reads from 15 diverse human tissues and cell lines [19], one lane of RNA-seq reads in human lymphoblastoid tissue [18] and another RNA-seq lane from CD4 cells [20]. In terms of genomic DNA based experiments, we analyzed five lanes sequencing the PhiX genome using varying platforms, reagents, and read lengths [21], one ChIP-seq lane mapping PAF binding sites in human CD4 cells [22], one ChIP-seq lane mapping CTCF binding sites in human embryonic cells [23], and one lane that served as a control in a ChIP-seq experiment in Jurkat cells [24]. For each dataset, we used only uniquely mapped reads, and further discarded reads with a mismatch at the first position of the read (see Materials and Methods). The latter criterion was set to ensure that the bias observed at the first position did not reflect sequencing errors.

**Figure 1:**
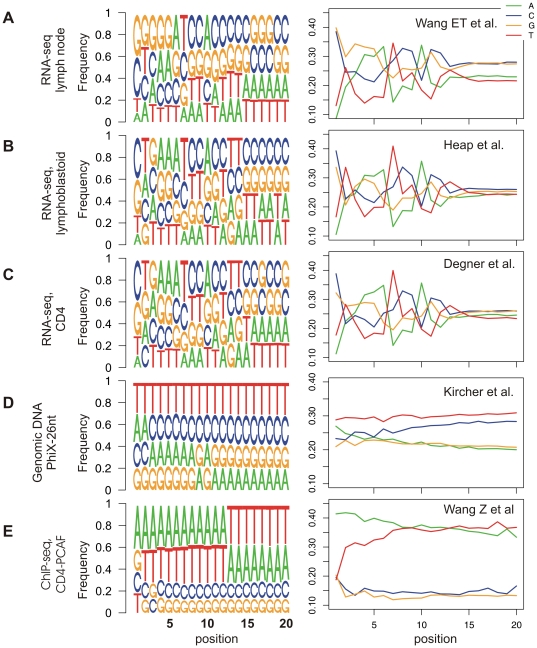
Examination of nucleotide biases within reads across different datasets of deep-sequencing experiments. For each dataset, we present sequence logos of the first twenty positions of all reads that could be aligned to the reference genome (left panel), and positional nucleotide charts (right panel). In the sequence logos, the height of each letter is proportional to the frequency of the corresponding base at the given position, and bases are listed in descending order of frequency from top to bottom. The positional nucleotide charts display the frequency of each base-pair at each position. Data for additional datasets is presented in Supporting Information S1. (A) Data for RNA-seq reads from human lymph node obtained from [19]. (B) Data for RNA-seq reads from human lymphoblastoid tissue obtained from [18]. (C) Data for RNA-seq reads from CD4 cells were obtained from [20]. (D) Data for genomic reads from PhiX control lanes following 26 cycles were from [21]. (E) Data for ChIP-seq lane mapping PAF binding sites in human CD4 cells were from [22].

For most of the analyzed data, biases in nucleotide composition were observed at the beginning of reads (Fig. 1 and Supporting Information S1), albeit of variable magnitudes. These biases were particularly strong at the first position of reads, but in some cases also extended into the subsequent positions. The biases were mostly present in RNA-seq, but were also present in one analyzed ChIP-seq data of Wang et al. (Fig. 1E). To assess whether these biases resulted from biased PCR amplification during the sequencing reaction, we repeated these analyses using only unique sequence tags, but the obtained results were practically indistinguishable, demonstrating that the effect cannot be attributed to biased PCR amplification (Supporting Information S1). To a limited extent, these biases can be attributed to random hexamer priming during reverse transcription (see Discussion). As demonstrated below, this bias can have a profound effect on downstream analyses.

### Mappability bias

In NGS data analysis, uniquely mapping reads are typically summarized over genomic regions. However, since genomic regions differ in terms of their sequence complexity, regions with lower sequence complexity will *a priori* tend to end up with lower sequence coverage than their more complex counterparts. Introns and exons constitute a good model based on which to study the effect of mappability, as they are both part of the same transcript, but the general sequence complexity of introns is expected to be reduced, compared to exons, since they are more dense in repetitive elements [25], [26].

**Figure 2:**
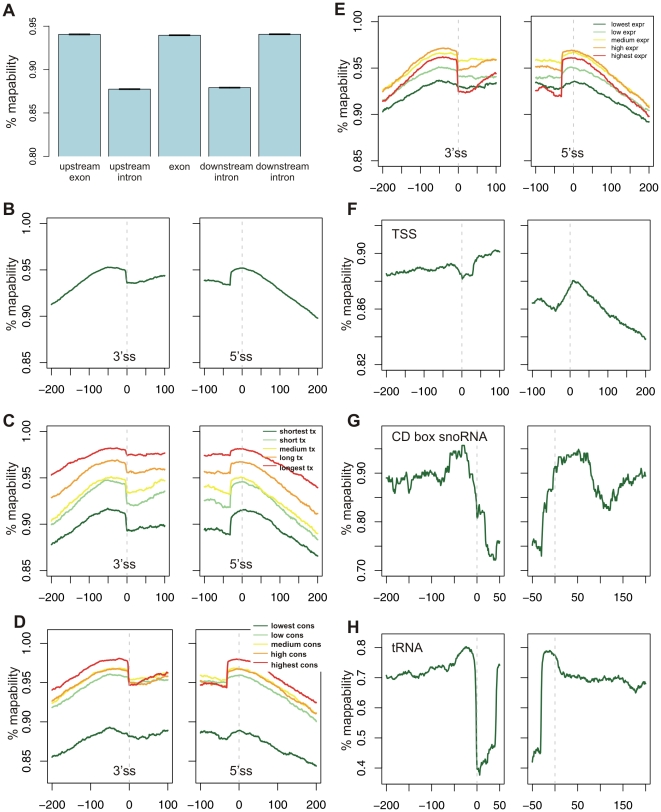
Mappability within genomic regions. (**A**) Mean mappability density values within internal exons and within the exons and introns flanking them. Error bars represent the standard error of the mean (SEM). (**B**) Mappability in the region surrounding exon/intron junction. The dashed line represents the exon/intron junction. (**C**) Mappability in the region surrounding exon/intron junction as a function of total transcript length. Each exon was distributed into one of five bins based on the length of the transcript containing it. (**D**) Mappability in the region surrounding exon/intron junction as a function of exon conservation level, divided into five bins. (**E**) Mappability in the region surrounding exon/intron junction as a function of transcript expression level, divided into five bins. Transcript expression levels were obtained from [27]. (**F**) Mappability in the regions surrounding transcription start and end sites. (**G**) Mappability in the regions surrounding CD box snoRNA start and end sites. (**H**) Mappability in the regions surrounding tRNA start and end sites.

To explore the impact of mappability in the context of exon-intron architecture in human, we generated genome-wide mappability maps of both strands, assuming reads lengths of 32 nucleotides. This was done by extracting the 32 nt sequence beginning at each genomic position, and mapping this sequence against the entire human genome. Sequences that could not be uniquely mapped to the genome were considered unmappable, and the genomic positions from which they originated were considered unmappable positions. We then constructed a dataset of 113,261 exon-intron quintets, each composed of an internal exon along with two flanking introns and two flanking exons (see Materials and Methods) and calculated mean mappability densities within the central exons and within the introns and exons flanking them (Fig. 2A). As expected, exons had significantly higher mappability levels than introns: mean mappability densities within exons were ∼94%, whereas within introns the mean densities were only ∼88% (Student's t-test, *P*<2.2e^−16^). To examine whether mappability levels were uniform within exonic and intronic regions, we examined mean mappability values within the genomic regions surrounding the two exon/intron junctions (Fig. 2B). This analysis confirmed the higher levels within exons and, surprisingly, also revealed a peak of mappability located in the intronic region adjacent to the two junctions, rather than within the exon itself (see Discussion).

Failure to eliminate the mappability bias will lead to increased read densities within regions with higher mappability. This can lead to spurious results, since subsequent analysis revealed that mappability was correlated with certain biological features. We began by examining mappability as a function of transcript length. We divided all exons into five bins based on the length of the transcripts comprising them and examined mappability across the region surrounding the exon/intron junctions within each bin. We found a positive correlation between transcript length and mappability (Fig. 2C). This association was highly significant (Kruskal-Wallis rank sum test, P<5.7e-227, see Materials and Methods). We next divided all exons into five bins based on evolutionary conservation levels of the exons among 18 placental mammals (see Materials and Methods). We observed a positive correlation between mappability and conservation with ∼10% differences in mappability between the most conserved and least conserved exons (Kruskal-Wallis rank sum test, P∼0) (Fig. 2D). Finally, we divided all exons into five bins based on transcript expression levels in lung fibroblasts obtained from [27] (this particular tissue was chosen as it was relevant for subsequent analyses). We again observed a clear, albeit more complex, relationship between the expression level and mappability, with the highest differential between exons and introns found in exons from highly expressed genes (Kruskal-Wallis rank sum test, P<5.8e-19) (Fig. 2E). Thus, without proper normalization of mappability, even if reads are uniformly and randomly simulated from the genome, exonic regions from long transcripts will have the highest read densities, more conserved exons would have greater densities, and exons from highly expressed genes would have the greatest differential between exon and introns in terms of read densities. These correlations with biological feature can be highly misleading, as they will lead to skewed results suggestive of representing biological phenomena. We highlight that these biases are only dependent on read length, but not on sequencing platform or type of experiment.

To further investigate the biases introduced by mappability, we examined mappability within genomic regions other than exon-intron boundaries. Specifically, we examined the regions surrounding start and end sites of transcripts (Fig. 2F), various non-coding RNA genes (Fig. 2G–H and Supporting Information S1), and coding sequence start and end sites (Supporting Information S1). In these cases, as well, mappability was far from being uniform. Such variation can profoundly impact the results of analysis of read stemming from these regions.

### Manifestation and normalization of the biases in the context of an NGS dataset

We originally encountered the two biases presented above, as well as two additional biases, in the analysis of a dataset mapping transcriptionally engaged polymerase in the context of exon-intron architecture. What initially appeared to be a striking biological phenomenon turned out to be the results of these biases. Below we detail this analysis, to exemplify how these biases are manifested, and what steps and controls we used to recognize and eliminate them.

Core et al devised and implemented a method termed global run-on sequencing, or GRO-seq, to map and quantify transcriptionally engaged RNA polymerases in a genome-wide fashion [28]. In this method, nuclear run-on assays (NRO) are employed to extend nascent RNAs that are associated with transcriptionally engaged polymerases under conditions in which new initiation is blocked. NRO-RNAs are subsequently isolated and subjected to next-generation sequencing. Thus, regions enriched in GRO-seq reads reflect regions enriched in transcriptionally engaged polymerase. Notably, in analyzing this data, we aimed to also address a specific research question, namely whether RNA polymerase II (RNAPII) kinetics decrease near splice sites. In light of the finding that exons are ‘marked’ by nucleosomes [29]–[35], we speculated that as RNAPII approaches an exon/intron junction, the presence of the nucleosome may reduce transcriptional rates [29], allowing time for the precise assembly of spliceosomal components over the exon/intron junction [36]–[39].

To detect whether exon-intron junctions were enriched in transcriptionally-engaged polymerase, we aligned a dataset of >23 million GRO-seq reads from lung fibroblasts to the human genome; we retained >11 million reads uniquely mapping the genome, similar to the number obtained by Core et al. [28]. As in [28], the 5′ most coordinate of the read was considered to reflect the position of transcriptionally engaged RNAPII. Each genomic position was allocated a score equal to the number of reads beginning at that position. We then used the above-constructed dataset of exon-intron quintets, and plotted the mean read densities in the regions surrounding the two ends of the exons, i.e., the 3′ and 5′ splice sites (3′ss and 5′ss respectively). Since the number of reads obtained from a given transcript is highly correlated with the expression level of the gene coding that transcript, we divided the exons into five equally sized groups based on the expression levels of the genes in which they are located; gene expression levels in lung fibroblasts were obtained from [27]. In our initial analysis we adopted a naïve approach, and did not take into account the nucleotide per cycle or the mappability biases.

**Figure 3:**
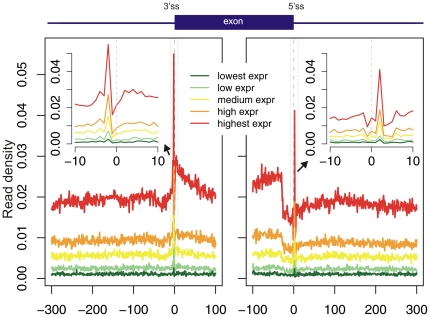
GRO-seq reads localization along exons and introns. Exons were aligned by their 3′ss (left panel) or by their 5′ss (right panel). The dashed line represents the exon/intron junction. Exons were divided into five bins based on microarray-based transcript expression levels in lung fibroblasts obtained from [27]. Insets present blowups of the regions marked by black rectangles.

Our analysis, presented in Fig. 3, revealed several phenomena: First, a prominent peak in GRO-seq reads was observed at each of the splice sites. Second, increased read densities were observed within exons, compared to introns. Third, decreased densities were observed within the terminal ∼30 exonic nucleotides with respect to the remaining exonic regions. These phenomena were present across all gene expression levels. The first phenomenon was suggestive of pausing of RNAPII at splice sites, while the second was suggestive of decreased transcriptional rates within exons compared to introns; However, subsequent analysis revealed that these results are due to the two above-described biases, combined with two more specific biases present in this particular dataset. We emphasize that in analyzing their data, Core et al. [28] made no claims in their manuscript pertaining pausing of RNAPII at splice sites or decreased rates within introns; Thus, the results presented here do not disprove the conclusion made by Core et al.

**Figure 4:**
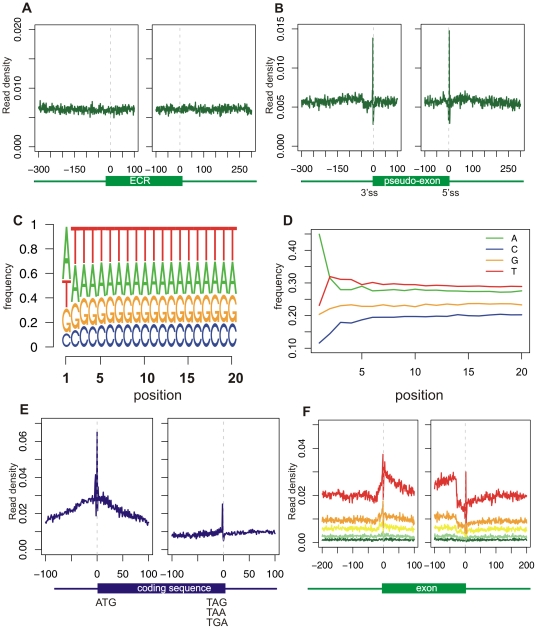
Control analyses of GRO-seq reads. (**A**) Analysis of 36,905 exonic compositions regions (ECRs) obtained from [32]. ECRs were defined as exon-sized region within intronic or intergenic regions with sequence content similar to that of exons, flanked by regions with intronic sequence content. (**B**) Analysis of 49,276 pseudo-exons obtained from [30]. Pseudo-exons were defined as regions with a length distribution similar to that of exons flanked by relative strong splicing signals. (**C**) Sequence logos of all aligned GRO-seq reads, aligned by their 5′ end, as in Figure 1. (**D**) Positional nucleotide charts for GRO-seq reads, as in Figure 1. (**E**) Alignment of GRO-seq reads in the 200 nt surrounding transcription start and end sites (left and right panels, respectively). (**F**) Analysis as in Figure 1 following normalization of all read counts by the relative frequency of the nucleotide at the first position of each read.

What initially suggested that the GRO-seq peak at the junctions reflected bias was the fact that it was replicated in a negative control. We made use of two such controls: The first was a dataset of 36,905 exonic compositions regions (ECRs) from [32]. ECRs were defined as exon-sized region within intronic or intergenic regions with sequence content similar to that of exons, flanked by regions with intronic sequence content. The second control was a dataset of 49,276 pseudo-exons [30], defined as regions with a length distribution similar to that of exons flanked by relative strong splicing signals. Examining the densities of GRO-seq reads in the regions flanking the two control sequence regions, we observed uniform read densities around ECRs (Fig. 4A); However, we found clear peaks around the pseudo-exons, similar to those observed around exons (Fig. 4B). These results suggested that the peaks at the exon/intron junctions were due to specific sequence biases present at the 5′ and 3′ splice sites. A closer inspection of the peaks over the exon/intron boundaries revealed that the peaks correspond to two single positions: position -2 of the 3′ss and position +3 of the 5′ss (Figure 3A, left and right insets, respectively). Since ‘A’ is the consensus nucleotide at both positions (***A***G at the 3′ss and GT***A*** at the 5′ss), we examined the distribution of nucleotides along the read positions within all aligned GRO-seq reads. We found that 42% of the reads began with ‘A’, whereas roughly 22% began with ‘G’ or ‘T’ and 12% with ‘C’ (Fig. 4C–D). Since we scored each genomic position based on the number of reads beginning at that position, a position beginning with ‘A’ will, *a priori*, have a two-fold increased score with respect to ‘G’ or ‘T’ and almost 4-fold increased score with respect to ‘C’, explaining the ∼2-fold peak observed at the splice sites. To further confirm the presence of this bias, we examined a dataset of 21,121 protein-coding regions. Protein coding regions invariably begin with an **A**TG start codon and terminate with a TAG/TAA/TGA stop codon. Since both regions are highly enriched in ‘A’s, we expected, and observed, an enrichment in terms of GRO-seq reads at both termini of the coding region (Fig. 4E).

We next statistically normalized for this nucleotide bias, by weighting each read based on the relative frequency of reads starting with the nucleotide at its first position (see Materials and Methods). Following this normalization scheme, the bias at the exon/intron junctions was substantially reduced (Fig. 4F, compare with Fig. 3). Since the bias was not completely eliminated, we explored the possibility of taking into account not only the first nucleotide of each read, but the combination of the first *k* nucleotides, where k>1 (see Materials and Methods). However, such normalization did not substantially alter the results (data not shown).

Following normalization for the nucleotide bias, the bias at the exon/intron junction was reduced, but not eliminated (Fig. 4F). We reasoned that the increased mappability within exons, with respect to introns, might impart additional bias. To remove the bias introduced by mappability, we identified all unmappable regions in both strand, and removed these positions from our calculations. Thus, density values were calculated as the sum of reads mapping within a region, divided by the number of mappable nucleotides within that region. We emphasize that this normalization was performed by Core et al. [28], and is therefore not expected to influence their results.

### Contamination with mRNA bias

**Figure 5:**
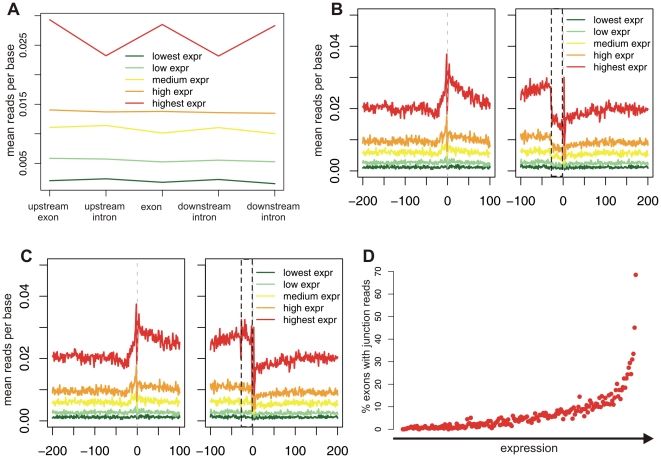
Analysis of effect of contamination of run-on experiment with mature RNA. (**A**) Mean number of GRO-seq read densities within exons and their flanking exons and introns as a function of expression levels obtained from [27], following normalization by mappability. (**B**) Analysis as in panel A, showing mappability values at a single base pair level. The dotted rectangle marks the region harboring the ∼30 terminal nt. (**C**) Analysis as in B, but incorporating reads obtained from exon-exon junctions. (**D**) Exons were divided into 200 equally sized bins based on gene expression levels derived from [27]. The percentage of exons with reads overlapping the junction between the central exon and the exon upstream to it are plotted for each bin.

Once mappability was accounted for, we expected read levels within exons to equal those within introns, as was reported by Core et al. [28]. However, levels within exons remained considerably higher than those within introns. This was particularly evident when all exons were divided into bins based on expression levels (Fig. 5A–B). Within the highest expression bin, there was a 22–23% increase in levels within the central exon, with respect to the two introns flanking it, and differences were highly significant (Student's t-test, *p* = 3.2e^−12^ and 2.4e^−12^ for upstream and downstream introns, respectively). In addition, even following normalization of both the nucleotide and the mapping biases, there still remained a considerable ‘valley’ over the ∼30 nt at the 3′ end of exons. The combination of the general increase in reads in exons relative to introns and of this valley led us to speculate that the dataset of GRO-seq reads was contaminated with mRNA. Such contamination would explain both phenomena. First, it would lead to higher levels of reads within exons, since introns are removed from the mRNA and are therefore not sequenced. Second, reads originating from the 3′ end of exons would not be aligned to the genome, since the reads originate from ligations of two exons and not from consecutive genomic regions.

To assess whether contamination with mRNA was a source of bias, we generated exon-exon libraries by concatenating adjacent exons and mapped all >23 million GRO-seq reads to these libraries. We retained only uniquely aligned reads that overlapped by at least 4 nt to each side of the exon-exon junctions and that either could not be aligned when we originally mapped them against the human genome or that had higher alignment scores when mapped to the junctions dataset than against the genome. We found 9,222 junctions between the central exon and the downstream one that were covered by at least one read. Following incorporation of the junction reads with the genomic reads, the valley at the 3′ end was essentially eliminated (Fig. 5C), indicating that indeed the valley originated from exon-exon concatenations. Since highly-expressed exons have ∼23% increased read densities compared with introns, this suggests that up to 23% of the GRO-seq signal within exons stems from contamination with mRNA (see Discussion). Examining this from a different perspective, we divided all exons into 200 bins of gradually increasing expression and calculated the percentage of exons with junction reads in each bin. Whereas the percentage of junctions in genes expressed at low levels was negligible, for highly expressed genes up to 70% of the exons were overlapped by junction reads (Fig. 5D). This, again, indicates that there were considerable levels of contamination with mRNA in the GRO-seq dataset.

**Figure 6:**
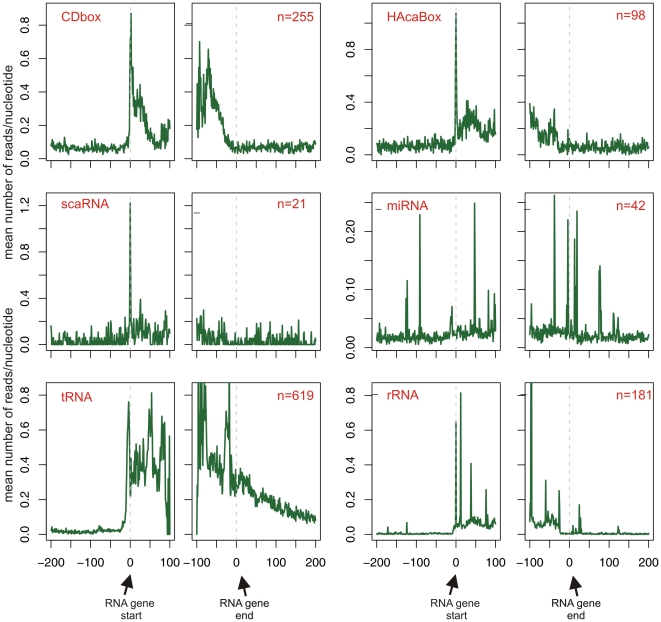
Plots showing GRO-seq read distribution in the along start and end sites of various non-coding RNA genes. The name of the RNA gene family and number of genes analyzed per family are indicated in red within the left and right panels, respectively.

As further evidence for contamination of the GRO-seq data with RNA not originating from nuclear run-on experiments, we examined read distributions along various non-coding RNA families. In particular, we were interested in examining box C/D and box H/ACA snoRNAs, both of which are responsible for modification of RNA molecules [40]–[42], and small Cajal body RNPs (scaRNPs), which direct modifications of snRNAs [43]. These RNA are encoded within introns of host genes and are released via post-transcriptional exonucleolytic trimming from the 5′ and 3′ ends of the debranched intron lariats [42], [44]. Thus, GRO-seq read levels of these molecules are expected to equal the background levels within the introns surrounding them, since their biogenesis occurs as part of the transcription of their hosting gene. However, we found that read densities within the bodies of the various RNA genes was between 5- and 10-fold higher than were read densities within the genes hosting them. This phenomenon is evident upon examination at the genomic regions surrounding the start and end sites of the RNA genes (Fig. 6). This phenomenon indicates that GRO-reads contain non-coding RNA sequences that did not originate from nuclear run-on sequencing.

### Non-hydrolysis bias

An additional prominent observation in Fig. 6 is the peak at the 5′ end of most analyzed RNA genes (Fig. 6). This peak is reminiscent of the peaks reflecting promoter-proximal pausing that occurs around transcription start sites of independently transcribed genes [28], [45]–[47]. One theoretical possibility is that this peak reflects pausing of RNAPII at the 5′ end of RNA genes of various families. However, since this peak is highly localized within the first nucleotides of snoRNA and scaRNAs, which are processed from within longer transcripts, we consider it likely that this finding results from bias as well. One of the first steps of the GRO-seq experiment is isolating and hydrolyzing run-on RNA; following reverse-transcription to cDNA, the 5′ ends of these RNAs are sequenced. Therefore, the first nucleotide at the 5′ terminus of coding transcripts or of RNA genes is biased to undergo sequencing, since no hydrolysis is required to obtain a read beginning at this position. This bias is presumably augmented by the fact that, as demonstrated above, the RNA pool is contaminated with mRNA and other non-coding RNA transcripts, leading to increased levels of 5′ termini in the sequenced pool.

## Discussion

In the years since deep-sequencing technology was launched, enormous efforts have been put into the development of platforms for mapping short reads and for downstream analysis of mapped reads, including assembly, identification of structure variants or SNPs, and detection of enriched regions (reviewed in [1], [2], [6]). In this study, we report several biases present in next-generation sequencing datasets. Notably, the two general biases we characterize – nucleotide per cycle bias and mappability bias – will mostly affect analyses in which two genomic regions are compared to each other, such as when exons are compared to introns or when gene bodies are compared to transcription start sites. They will probably have only a minor effect when examining the same region (e.g., gene/exon) under different experimental conditions, since that region will be affected by the bias in a similar manner in the different conditions.

Previous studies have found some links between nucleotide composition and various aspects of next-generation sequencing. Several studies have found that GC content correlates positively with read coverage [14], [15]. In addition, an association between erroneous nucleotide calls and nucleotide composition was found, with erroneous nucleotide calls considerably more likely to be flanked by ‘G’ nucleotide [14]. These studies also reported that the frequency of erroneous calls is increased at the first position, lowest at the second position, and the highest error rate is observed at the last positions of the read [14], [15]. However, the nucleotide-per-cycle bias reported here does not reflect sequencing error, since the bias is obtained also when examining only reads with full identify with the reference genome. Rather, this bias reflects sampling bias at some step of the sequencing reaction. While this study was being prepared, two different studies reported the prevalence of biases across the first positions of reads [16], [17], as reported here. Both studies focused mainly or exclusively on RNA-seq experiments. Hansen et al. concluded that the bias is mainly due to random hexamer priming [16]; however, Hansen et al. also noticed small biases in the first nucleotides of reads in ChIP-seq datasets [16], hinting that there might be additional sources for the observed bias. Indeed, in one of the ChIP-seq experiments analyzed by us, we observed very high levels of bias across the first positions (Fig. 1E), and we also observed a bias in the GRO-seq dataset, the protocol of which does not include priming by random hexamers. Therefore, the sources of these biases remain to be determined.

Although studies are increasingly incorporating mappability into their analyses (e.g., [28], [48]–[52]), this remains far from common practice. Considering mappability is particularly crucial in the comparison between regions such as exon and introns. Over the past year, various groups have used NGS reads from different experiments to compare exons to introns in different contexts. Several studies, including one by us, found increased levels within exons in terms of nucleosome occupancy and specific post-translational histone modifications [29]–[33], [35], [53]. However, with the exception of one study [32], data was not normalized by the differential mappability between exons and introns. Other studies, also based on NGS technology, have found differential levels of DNA methylation within exons compared with introns [10]–[12]; none considered mappability in their analyses. As shown here, mappability correlates with various biological features, such as transcription length and expression levels. Therefore, changes originating in mappability may falsely present themselves as biological phenomena. This notwithstanding, in our analysis the difference in mappability of exons compared with introns was 8%; Thus, fold-changes substantially larger than this cannot be attributed to mappability alone.

Notably, mappability is not important only in the context of exon-intron regions. First, as shown in Fig. 4, many genomic regions of interest have distinct mappability profiles and normalization must be used to omit this inherent bias. When RNA-seq experiments are used to infer gene expression levels or inclusion levels of exons, mappability corrections must be made to avoid bias [48], [52]. Similarly, in ChIP-seq analysis in which reads from both strands are typically aggregated and compared to a background or empirical model, assessments are bound to be skewed due to failure to account for mappability. In this context, many tools exist for ChIP-seq analysis, and some of them allow incorporation of mappability as a global parameter [54]–[56]. However, these tools essentially assume that mappability is uniform across the genome, which is far from being the case. To our knowledge, the only tool for ChIP-seq analysis which takes into account regional mappability is PeakSeq [54].

Considerably complicating incorporation of genome-wide base-by-base mappability into analysis of NGS reads is the fact that such maps must ideally be specifically tailored based on read length, alignment algorithm and alignment parameters, since these parameters will all influence whether a read will be considered mappable. Pre-prepared maps exist for certain read lengths (e.g., the ‘Mappability’ tracks in the UCSC genome browser, http://genome.ucsc.edu/). However, since each specific experiment will generate reads of a specific length and use specific alignment parameters, these pre-prepared maps will not give a precise picture of the mappability for that specific experiment. To obtain such maps one is currently forced to separately align ∼3 billion reads (each genomic position) against the entire human genome. This is time intensive and requires extensive computational resources. As this may be prohibitory for laboratories lacking the required facilities, tools efficiently addressing this issue are desired.

The two biases discussed above are relevant for the vast majority of NGS experiments, regardless of whether they are aimed at DNA or RNA. In contrast, the bias of non-hydrolysis is mostly relevant for analyses at the RNA level (e.g., RNA-seq, or GRO-seq), whereas the mRNA contamination bias is specifically relevant for GRO-seq experiments or other experiments at the pre-mRNA level. We found that contamination of GRO-seq reads with reads due to mRNA and non-coding RNAs was ∼23%, based on the differences between read levels in exons compared with introns. This is considerably higher that the estimate by Core et al. [28], who calculated that the purity of the nuclear run on RNA pool in their experiment was ∼98%. This discrepancy is to some extent resolved once it is considered that introns are longer than exons by at least one order of magnitude. Consider a theoretical model in which introns are 10-fold longer than exons and 100,000 nuclear run-on reads are generated uniformly. In this hypothetical experiment, ∼9,000 reads will result from exonic regions and ∼90,000 from intronic regions. If 2,000 contaminating mRNA reads (2%), originating exclusively from exonic sequence, are added to the pool, the exonic reads increase to ∼11,000, thereby increasing densities within exons by ∼20%, whereas coverage within intronic regions will remain essentially unchanged. Thus, a 2% contamination can lead to a 20% difference in read densities within exons; this difference can be higher depending on the exon to intron length ratio within the sequenced regions, or lower, depending of the fraction of contamination originating from mRNA, as opposed to tRNA and rRNA.

Finally, it is important to note that although all datasets analyzed in this study are based on Illumina technology, this is not expected to affect most biases presented here. Mappability does not change as a function of sequencing technology, but only as a function of read length and the genome the reads are mapped against; And non-hydrolysis and contamination with pre-mRNA are independent of platform as well since they precede the step of deep-sequencing. The only bias which may to some extent be platform dependent, is the nucleotide-per-cycle bias, the source of which partially remains to be determined; But at least for RNA-seq experiments, this bias appears to be present in additional platforms as well [16], [17].

## Materials and Methods

### Illumina NGS datasets

Twenty-five Illumina sequencing lanes were obtained from the studies detailed in the Results section. For all lanes excluding those of Wang ET et al., we used novoalign version 2.5 to align the reads against the relevant reference genome, using default parameters and ‘-t 73’ to allow a maximum of two mismatches. Using Perl scripts we then parsed the results to obtain reads that (1) aligned uniquely against the genome and (2) lacked a mismatch in the first position. For the lanes from Wang ET et al., we downloaded the aligned reads from [19] and parsed them even more stringently, not allowing a mismatch at any position. This was done to ensure that the strong bias observed throughout the first positions of all lanes in this dataset did not reflect sequencing errors.

### Dataset of internal exons and introns

Coordinates of human (hg18) genes were downloaded from the UCSC Genome Browser website knownGenes table. Based on this annotation, a dataset of exonic coordinates was generated. Each entry in this dataset consists of genomic coordinates of a central exon, the two exons flanking it, and the two introns lying in between. Redundant central exons sharing the same set of genomic coordinates were removed. Due to RNAPII buildup at the beginning and end of genes, we demanded that the exons upstream and downstream of the central exon be internal exons as well. To further ensure that reads along exons did not originate from promoter regions or transcription end sites, we removed all entries in which the central exon was within 1 kb of any annotated transcription start or end site, based on annotations in the knownGenes table. Moreover, we excluded any entry in which any of the quintet's exons or introns overlapped the coordinates of an RNA gene (see below), as these were analyzed separately. Finally, due to subsequent analyses of junctions, we also filtered out sets that contained exons or introns shorter than 32 nt; this filtered out only a negligent fraction of the original entries. Our final dataset contained 113,261 quintets. In analyses in which we compared read densities across the three exons in the quintets, we applied an even more rigid filter and demanded that the upstream exon not be within 1 kb of any transcription start site; this left 102,681 exons.

### Dataset of transcripts, coding sequences, and RNA genes

Datasets of 26,571 regions undergoing transcription and 21,121 protein-coding regions were obtained based on the knownGenes table. To avoid redundant analyses due to different isoforms mapping to similar genomic coordinates, for each set of transcripts/coding regions sharing a common clusterID, which was extracted from the knownIsoforms table, we retained only one isoform that was selected randomly. Coordinates of 7,118 RNA genes were obtained by merging two UCSC Genome Browser tables: the sno/miRNA table, which provides data on C/D and H/ACA box snoRNAs, scaRNAs, and microRNAs based on snoRNABase and miRBase [57]–[59], and the RNA genes table from which we extracted lower confidence RNAs, including snoRNAs, transfer RNAs (tRNAs), ribosomal RNAs (rRNAs), and others. The latter table also contains predictions of different RNA genes based on sequence similarity. Redundant entries were detected based on identical genomic coordinates and only the entry from sno/miRNA table was retained. In the sno/miRNA table, snoRNA genes are divided into three families: C/D box snoRNAs, H/ACA box snoRNAs, and scaRNAs. In the RNA genes table, there are no divisions into families of snoRNA genes. In our analyses, we separately analyzed RNAs originating from each of the two tables, retaining the original annotations.

### Normalization of nucleotide per cycle bias

To statistically enforce a uniform distribution of nucleotides at the first position of reads, we weighted each read based on the inversed frequency of reads beginning with the first nucleotide of that read. Since in subsequent steps we were interested in looking not only at the first nucleotide but at the regions of length *k*, or *kmer*, we gave each genomic position (*pos*) a *score*, as follows:
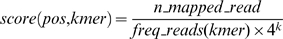
where *kmer* is a sequence of length *k* beginning at position *pos*, *n_mapped_reads* indicates the number of reads uniquely mapping to position *pos*, and *freq_reads* is the frequency of reads beginning with *kmer* within the total pool of reads. This scoring method artificially increases the score of reads beginning with rare *kmers*, and decreases the score of reads beginning with common ones.

To quantitatively assess to what extent normalization decreased the peak, we examined the fold change in the peak in GRO-seq reads in positions −2 and +3 with respect to the intronic background level of reads. As intronic background for position −2, we used the mean level of reads in position −200 to −100 of the upstream intron. As intronic background levels for position −3, we used the mean level of reads in positions +100 to +200 of the downstream intron. Calculations were performed separately for each of the five expression level bins.

### Mappability assessment

To determine genome-wide mappability, we exhaustively enumerated the hg18 reference genome sequences to generate genome-wide mappability data resources that profiled the extent to which 32-nt DNA sequences could be uniquely aligned to the genome using precisely the same parameters as above. Each 32-nt sequence that could not be uniquely aligned to the genomic region from which it originated was considered unmappable and its first position was considered an unmappable position.

### Analysis of mapped GRO-seq reads

We obtained >23 million short reads from two independent GRO-seq experiments within lung fibroblasts (IMR90) performed by Core et al. [28]. In light of the high correspondence between the two experiments, we pooled together the reads from the two experiments to yield greater statistical power in the subsequent analyses. We first trimmed the reads to 32 nt. Next, using the NovoCraft alignment software (http://www.novocraft.com/), we uniquely mapped 11,878,891 of the reads to the genome. We used the above-described parameters that did not allow more than two mismatches between the read and the genome and allowed only very minor tolerance to insertions/deletions. As in [28], the 5′ most coordinate of the read was considered to reflect the position of transcriptionally engaged RNAPII. To calculate the read densities within genomic regions (genes/exons), we summarized the number of reads occurring within a given genomic start and end coordinates and divided this number by the length of the interval between the two coordinates.

### Alignment of GRO-seq reads to exon-exon and exon-intron junctions

To align reads to exon-exon junctions, we created datasets of all exon-exon junctions in our datasets, by concatenating the 31 nt from the 3′ of a given exon with 31 nt from the 5′ of the exon immediately downstream to it. We generated two such datasets: One for the junctions between the upstream exon and the central one and one for the junctions between the central exon and the exon downstream to it. We next separately aligned each of the >23 million short reads against each of the two junction datasets, using the same parameters as in the alignment against the entire genome. We retained only aligned reads that overlapped by at least 4 nt to each side of the exon-exon junctions; this criteria was set to ensure that the reads originated from the junctions. All reads which uniquely mapped to the junctions were then compared with their mapping to the genome. We retained all reads that uniquely mapped to the junction dataset or that had a lower scoring alignment when aligned against the entire genome than the junction dataset.

### Gene expression levels

Gene expression levels were obtained from two expression arrays in lung fibroblasts [27]. The expression levels of the genes were averaged across the two samples and subsequently log-transformed to yield the expression level estimates of the genes.

### Conservation

Conservation measures for specific positions within the genome were assessed based on phastCons scores for 18 placental mammals, which were downloaded from the UCSC Genome Browser (http://hgdownload.cse.ucsc.edu/goldenPath/hg18/phastCons28way/placental/). This dataset includes a score for each nucleotide, ranging from 0 to 1, that provides a measure of conservation level of that nucleotide among placental mammals. Values were averaged over the 50 terminal intronic nucleotides to yield a mean conservation level of that region.

### Statistical tests

To examine the significance of the correlations between mappability and transcript length, expression levels, or evolutionary conservation (Figure 2C–E), we separately each of the following genomic regions: the 200 intronic nt upstream of exons, the first 100 nt of exons, the terminal 100 nt of exons, and the 200 intronic nt downstream of exons. For each of these regions, we established a distribution of mean mappability values. These were divided into five bins, as shown in the manuscript, and a kruskal-wallis rank sum test, which is a non -parametric one way analysis of variance, was calculated for each of the four regions. The P value quoted in the manuscript is the highest, i.e. **least** significant P value, of all regions.

## Supporting Information

Supporting Information S1 (PDF)
