# Supplementary material for: Detection and Removal of Biases in the Analysis of Next-Generation Sequencing Reads
Source: PLoS One. 2011 Jan 31;6(1):e16685. doi: 10.1371/journal.pone.0016685 (PMC3031631; doi:10.1371/journal.pone.0016685)
Supplement: Supporting Information S1 — (PDF) [file pone.0016685.s001.pdf]

Supplementary Materials:
Detection and removal of biases in the analysis of next-generation sequencing reads

Schraga Schwartz[1,2], Ram Oren[1,2], and Gil Ast[1]

[1]Department of Human Molecular Genetics and Biochemistry, Sackler Faculty of Medicine, Tel-Aviv University, Ramat Aviv 69978, Israel

[2]These authors contributed equally.

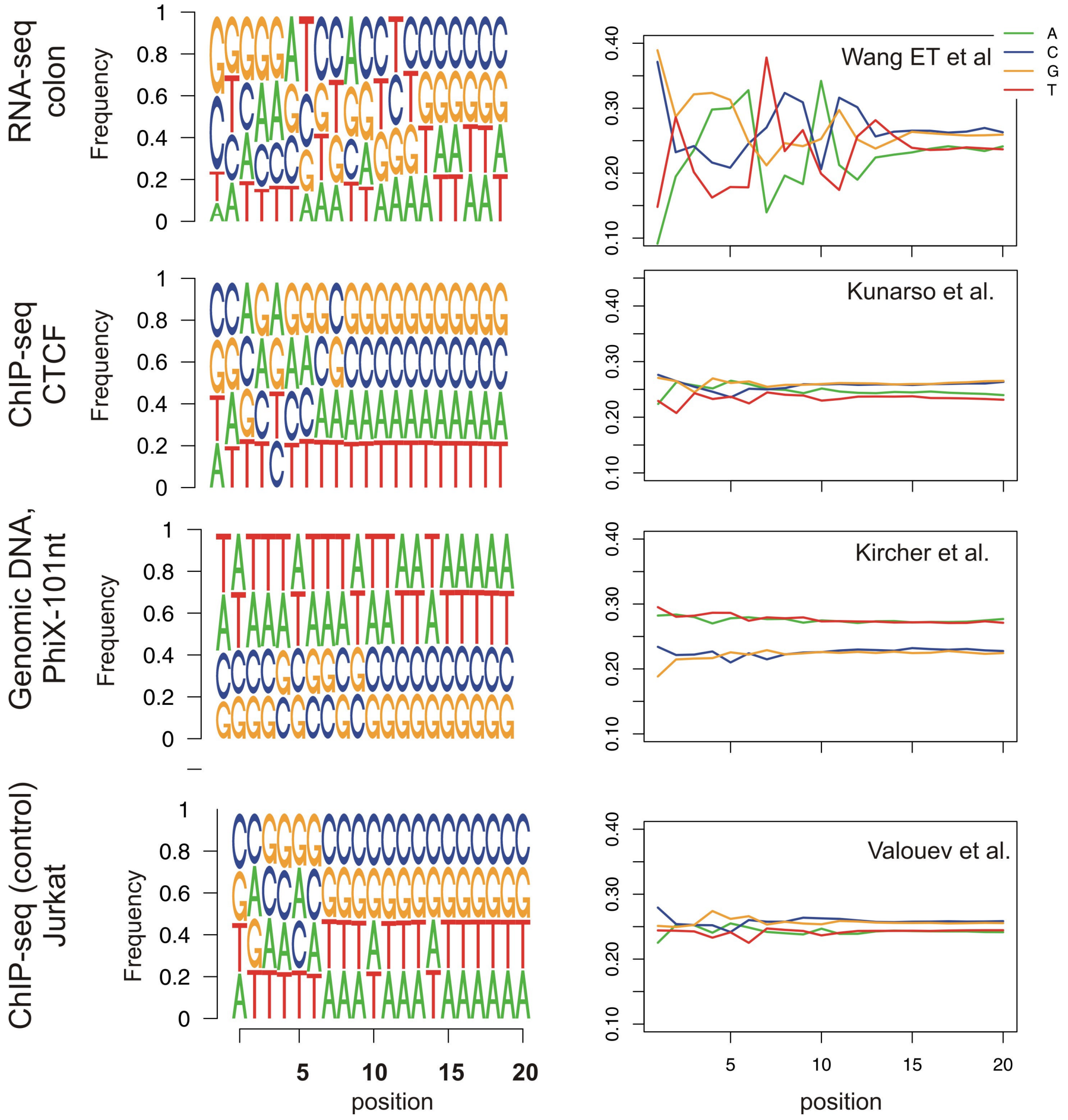

Supplementary Figure 1: Sequence logos and positional nucleotide plots for several additional examined datasets, as in Figure 1.

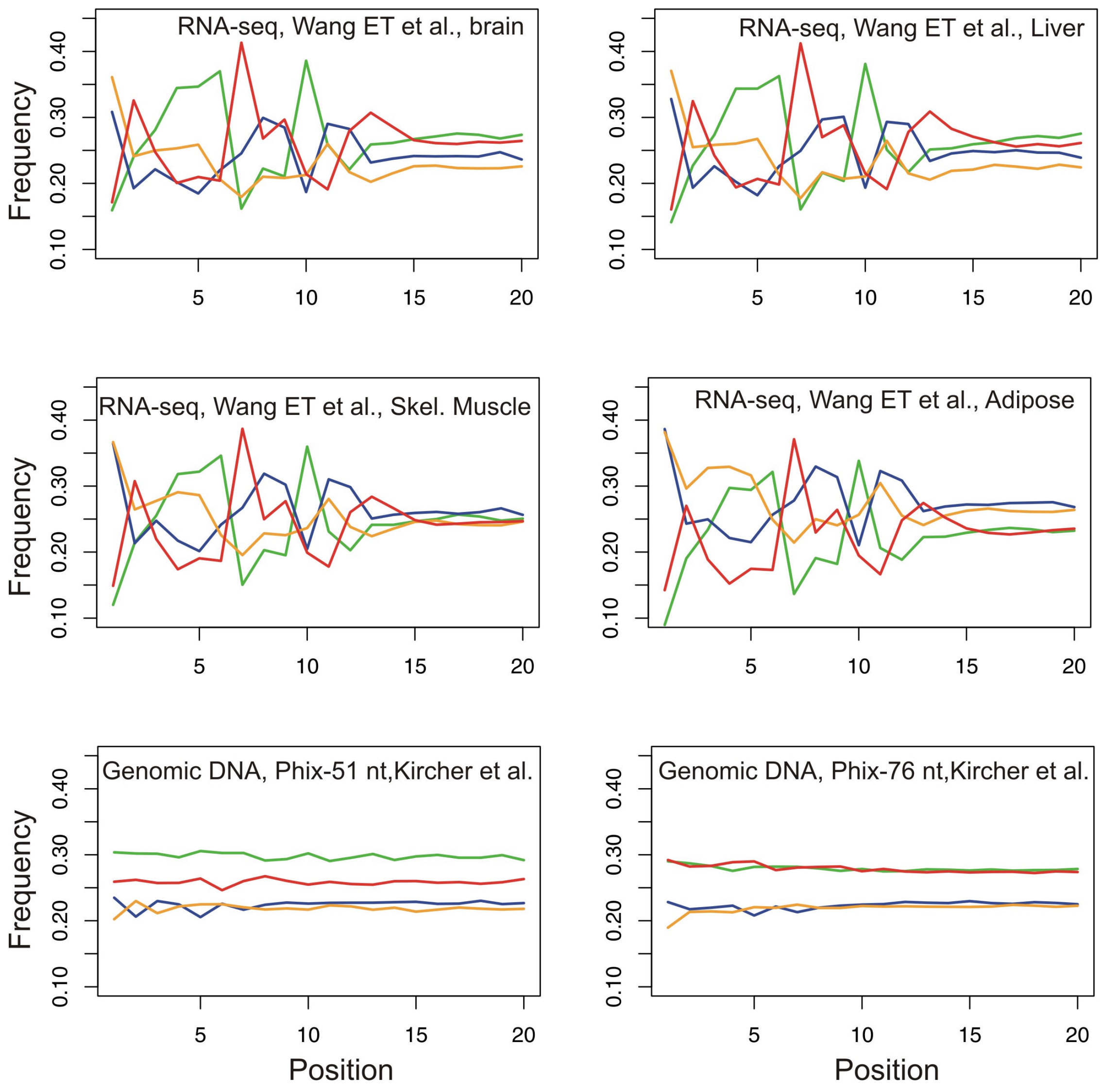

Supplementary Figure 2: Positional nucleotide plots for several additional examined datasets, as in Figure 1 and in Supplementary Figure 1.

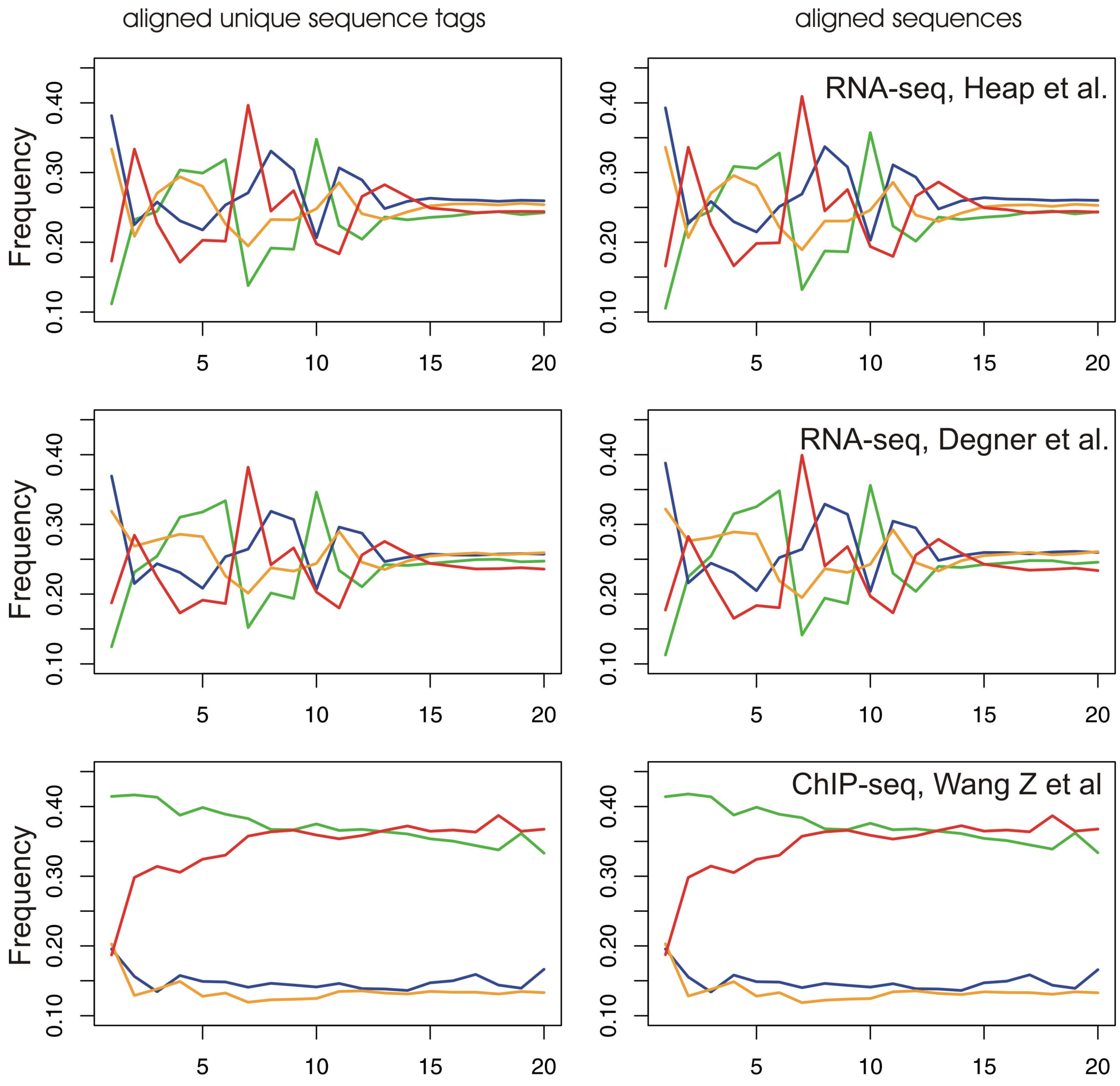

Supplementary Figure 3: Comparison of nucleotide composition when examining only unique sequence tags (left column), as opposed to all sequences (as shown in Figure 1 in the manuscript). This analysis demonstrates that the bias in nucleotide composition is not likely to have occurred as a result of biased PCR amplification of few specific amplicons.

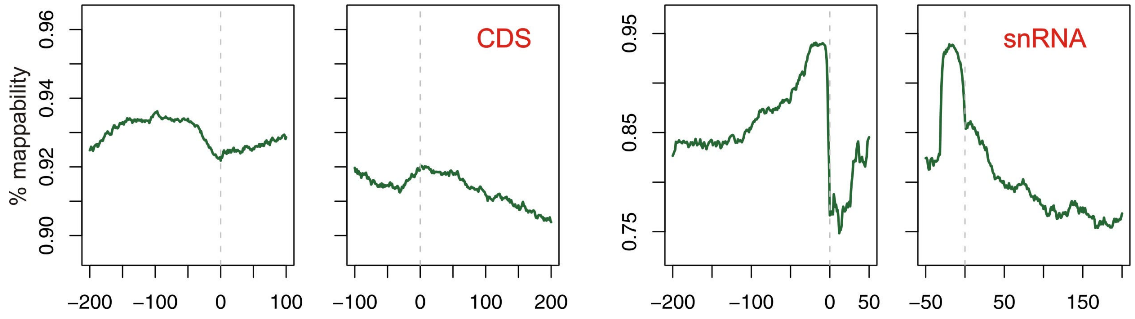

Supplementary Figure 4: Mappability density values within the regions surrounding the coding sequence start and end sites, and snRNAs.

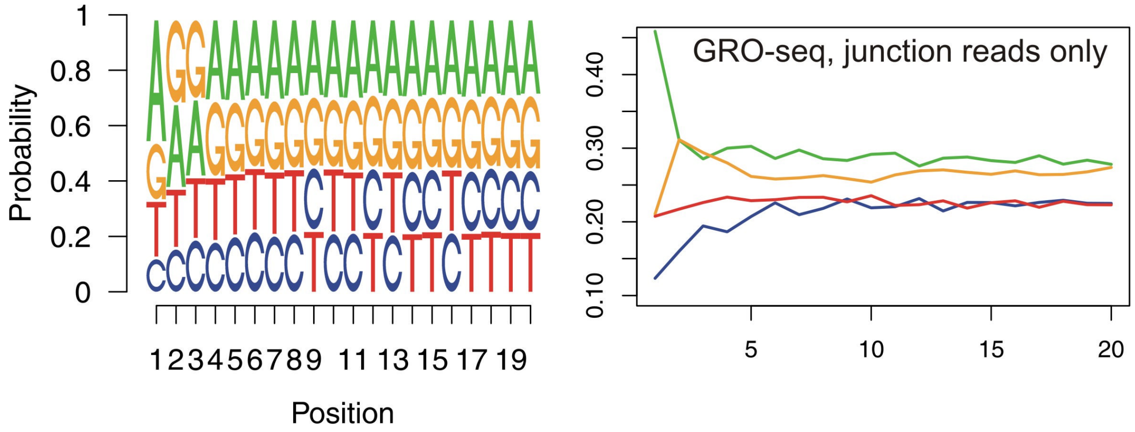

Supplementary Figure 5: Sequence logos and positional nucleotide plots for junction reads in GRO-seq data. The similarity of these plots to the ones in Figure 4C-D demonstrates that the bias is presumably not due to biased hydrolysis of BrU containing residues, as it is present also in reads originating from contaminating mRNA.
